# The Role of MAPK12 in Prognosis of Patients With Liver Cancer and Effects on Stemness Characteristics

**DOI:** 10.1155/sci/9071464

**Published:** 2025-08-27

**Authors:** Yun Tao, Jie Tang, Wenhui Yu, Wenge Yang, Jianwei Zhang, Qinghua Wu, Jie Li

**Affiliations:** Department of Interventional Radiology, Affiliated Hospital of jiangnan University, Wuxi, China

**Keywords:** hepatocellular carcinoma, machine learning, prognosis, stemness

## Abstract

Liver hepatocellular carcinoma (LIHC) is a prevalent and highly aggressive form of liver cancer, characterized by increasing rates of incidence and mortality globally. Although numerous treatment options currently exist, they frequently result in insufficient clinical outcomes for those diagnosed with LIHC. This highlights the urgent need to identify new biomarkers that can enhance prognostic evaluations and support the development of more effective therapeutic strategies for LIHC. Through the use of the SwissTargetPrediction tool, we precisely identified molecular targets related to Sorafenib. Furthermore, analysis of RNA sequencing data from the TCGA-LIHC cohort uncovered 24 genes associated with different patient prognoses following Sorafenib therapy. Employing a clustering-based analytical approach, we assessed the connections between gene expression profiles, clinical outcomes, immune cell infiltration levels, and tumor stage progression. A prognostic framework was constructed by applying various machine learning techniques and subsequently validated across several independent datasets. Utilizing the XgBoost algorithm, MAPK12 emerged as a key regulatory gene influencing the prognosis of individuals with LIHC. The results of in vitro experiments demonstrated that knockdown of MAPK12 reduced the proliferation, metastasis, and tumor stemness-related sphere-forming ability of LIHC cells. These results underscore the promise of MAPK12 as a potential prognostic biomarker for LIHC and offer valuable insights for crafting personalized treatment approaches.

## 1. Introduction

Liver hepatocellular carcinoma (LIHC) ranks as one of the most common malignant tumors worldwide, with recent reports indicating increasing morbidity and mortality rates [1–4]. Although a range of treatment approaches exists—including surgical resection, local ablation, and systemic therapies—patient outlooks remain generally poor. This is largely due to the pronounced heterogeneity of LIHC and the intricate characteristics of its tumor microenvironment [5]. Current immune-related markers employed for prognostic purposes in LIHC face notable limitations because their expression can vary substantially among individuals and often fail to capture the complex dynamics within the tumor's immune microenvironment. Consequently, these biomarkers are insufficient for effectively guiding personalized immunotherapy regimens. Additionally, many LIHC patients suffer from worsening liver function alongside other comorbidities, which further challenge the successful implementation of immunotherapy [6]. Therefore, it is critical to discover novel biomarkers that have the potential to improve prognostic predictions and ultimately enhance patient outcomes.

Sorafenib, an orally administered multitarget tyrosine kinase inhibitor, is widely employed in managing LIHC [7–9]. This drug works by disrupting tumor cell signaling pathways through the inhibition of several tyrosine kinases involved in cell proliferation and survival [10]. In particular, sorafenib targets Raf kinase, thereby, impacting the MAPK/ERK signaling cascade, which is crucial for cellular growth and survival [11]. The MAPK pathway holds a central role in LIHC, with various components performing distinct functions during tumor initiation and progression. For instance, ERK1 primarily mediates cisplatin-induced apoptosis, while inhibiting ERK2 leads to increased ERK1 activity, thereby, enhancing cancer cells' responsiveness to cisplatin treatment [12]. These findings suggest that selective targeting of ERK2 might boost cisplatin's effectiveness by amplifying ERK1′s proapoptotic actions in LIHC therapy. Moreover, the S100A9 protein promotes LIHC cell proliferation and invasion by activating the RAGE-dependent ERK1/2 and p38 MAPK pathways. Studies have shown that the interaction of S100A9 with RAGE triggers the MAPK signaling cascade, fostering tumor growth, and invasiveness. Thus, S100A9 and its related pathways represent promising therapeutic targets in LIHC [13]. Overall, the MAPK signaling network plays diverse roles in LIHC development. Further investigation of its precise mechanisms may provide novel insights and therapeutic strategies for this cancer type. Sorafenib effectively suppresses tumor cell growth and division by acting on these signaling pathways. Additionally, it blocks tumor angiogenesis by inhibiting vascular endothelial growth factor (VEGF) and its receptors [14]. Because angiogenesis is essential for tumor progression and metastasis, sorafenib impairs tumor advancement by disrupting blood vessel formation and restricting nutrient supply to the tumor. Beyond this, sorafenib modulates immune cell function within the tumor microenvironment, enhancing antitumor immune responses. Recent data suggest that combining sorafenib with memory-like natural killer (NK) cell immunotherapy could yield improved treatment outcomes in LIHC [15]. Clinical trials have confirmed that sorafenib significantly extends survival in advanced LIHC patients, securing its role as a standard therapeutic option [16]. However, new evidence indicates that sorafenib's effectiveness varies among individuals, underscoring the need for more research on its molecular targets to better predict patient responses and optimize immunotherapy strategies. These insights pave the way for tailored treatment approaches in LIHC management.

This study aimed to explore the role of sorafenib-related genes in predicting prognosis and immunotherapy response in LIHC patients. The molecular structure of sorafenib was first acquired from the PubChem database, followed by the identification of its potential targets using the SwissTargetPrediction tool [17]. Through analysis of the TCGA-LIHC dataset, we discovered 24 genes linked to varied prognostic outcomes in the context of sorafenib treatment. We then conducted cluster analysis based on the expression profiles of these genes to examine their associations with patient survival, immune cell infiltration, and tumor pathological stage. Moreover, clinical information from the TCGA-LIHC cohort was employed to establish a prognostic model applying multiple algorithms, and its performance was validated with external datasets, including GSE76427 and ICGC. Using the XgBoost machine learning algorithm, MAPK12 was identified as a critical regulatory gene connected to sorafenib's effects. Lastly, in vitro experiments confirmed that knockdown of MAPK12 diminished stemness properties in LIHC cells.

## 2. Materials and Methods

### 2.1. Datasets and Patient Samples

Targets related to sorafenib were identified through the SwissTargetPrediction platform. In this study, we combined RNA sequencing data with clinical information obtained from the TCGA-LIHC dataset [18, 19]. To build and validate several prognostic models, multiple datasets were employed, including TCGA-LIHC, GSE76427, and ICGC. Specifically, the TCGA-LIHC cohort consists of 371 patients, encompassing a range of pathological stages and tumor grades; the GSE76427 dataset includes 115 LIHC samples; and the ICGC dataset contains clinical data from 240 patients based in Japan.

### 2.2. Negative Matrix Factorization (NMF) Cluster Analysis in TCGA-LIHC Dataset

We applied the NMF algorithm to detect biologically meaningful coefficients within the gene expression matrix, reorganizing both genes and samples to emphasize the underlying structural features of the dataset and aid in grouping samples [20]. For differential expression analysis, clusters A and B were compared using the 'Limma' R package, with thresholds set at |logFC| > 0.5 and an adjusted *p*-value below 0.05. Following this, all samples were grouped based on the differentially expressed genes identified from the subclusters. To explore possible molecular subtypes, the “NMF” R package was utilized, implementing the “brunet” method for 100 iterations per specific value, exploring cluster numbers ranging from 2 to 10. The ideal cluster count was selected by evaluating cophenetic correlation, dispersion, and silhouette width metrics.

### 2.3. Correlation Analysis of Immune Infiltration and Immunotherapy

To ensure a reliable assessment of immune scores, we utilized the immunedeconv R package [21]. Each algorithm within this package has undergone rigorous testing and offers unique advantages.

### 2.4. Building Prognostic Models

To develop a robust and precise prognostic model for LIHC, we applied multiple machine learning algorithms in various configurations. The TCGA-LIHC dataset served as the training set, while validation was performed using the GSE76427 and ICGC datasets. Each algorithm combination was assessed based on the area under the curve (AUC) metric, and the set yielding the highest average AUC was selected as the optimal prognostic model [22, 23].

### 2.5. Molecular Docking and Functional Analysis

Molecular docking was performed using the CB-Dock2 database, with the 3D structure of sorafenib sourced from the PUBCHEM database [24, 25]. To further elucidate the functions of potential targets, we conducted a functional enrichment analysis [26].

### 2.6. Cell Culture

In this study, two hepatocellular carcinoma cell lines, HepG2 and HCCLM3, were utilized. Authentication of all cell lines was performed using STR analysis, and they were verified to be free from mycoplasma contamination. The cells were maintained in a humidified incubator set to 37°C with 5% CO2.

### 2.7. qRT-PCR

Cells were treated with Trizol reagent to extract RNA, followed by reverse transcription to synthesize cDNA using the RevertAid First Strand cDNA Synthesis Kit. Quantitative real-time PCR (qRT-PCR) was then conducted on the Applied Biosystems 7900HT Fast Real-Time PCR System, according to the reaction setup detailed below. The primer sequences used were: for the target gene MAPK12, Forward: CAGGCAAGACGCTGTTCAAG and Reverse: TGGTCAGGATAGAGGCAAAATC; for the reference gene GAPDH, Forward: CGGAGTCAACGGATTTGGTCGTAT and Reverse: AGCCTTCTCCATGGTGGTGAAGAC.

### 2.8. Colony Formation Assay

The cells were placed in 6-well plates and incubated for a duration of 2 weeks. Following this incubation period, methanol was utilized to fix the cells, which were then treated with a 0.1% crystal violet solution for staining. An Olympus microscope was used to observe and count the cells [27].

### 2.9. Transwell Assay

Cell motility and invasion were examined using Transwell chambers, with some assays incorporating Matrigel (Corning, Inc.) and others performed without it. Briefly, 2 × 10^4^ transfected colon cancer cells were suspended in 100 µL of serum-free medium (Gibco; Thermo Fisher Scientific, Inc.) and added to the upper chamber. Meanwhile, 500 µL of DMEM supplemented with 10% fetal bovine serum (Shanghai ExCell Biology, Inc.) was placed in the lower chamber. The chambers were incubated at 37°C in a 5% CO2 atmosphere for 24 h. After incubation, cells were fixed using 4% paraformaldehyde (Beyotime Institute of Biotechnology) for 10 min at room temperature. Subsequently, cells were stained with 0.2%–0.5% crystal violet solution (Sigma–Aldrich; Merck KGaA) for 10 min at room temperature and then observed under an inverted optical microscope (Shanghai Optical Instrument Factory) for quantitative analysis. The migration assay followed the same procedure as the invasion assay, except Matrigel was not used in the upper chamber.

### 2.10. Statistics

The comparison between two groups was conducted using either student's *t*-test or a paired *t*-test. For all other variables, one-way ANOVA was performed along with Tukey's test. Pearson's correlation coefficient was utilized to analyze correlations. A significance level of *p* < 0.05 was established.

## 3. Result

### 3.1. Identification of Sorafenib Targets in Hepatocellular Carcinoma

**Figure 1:**
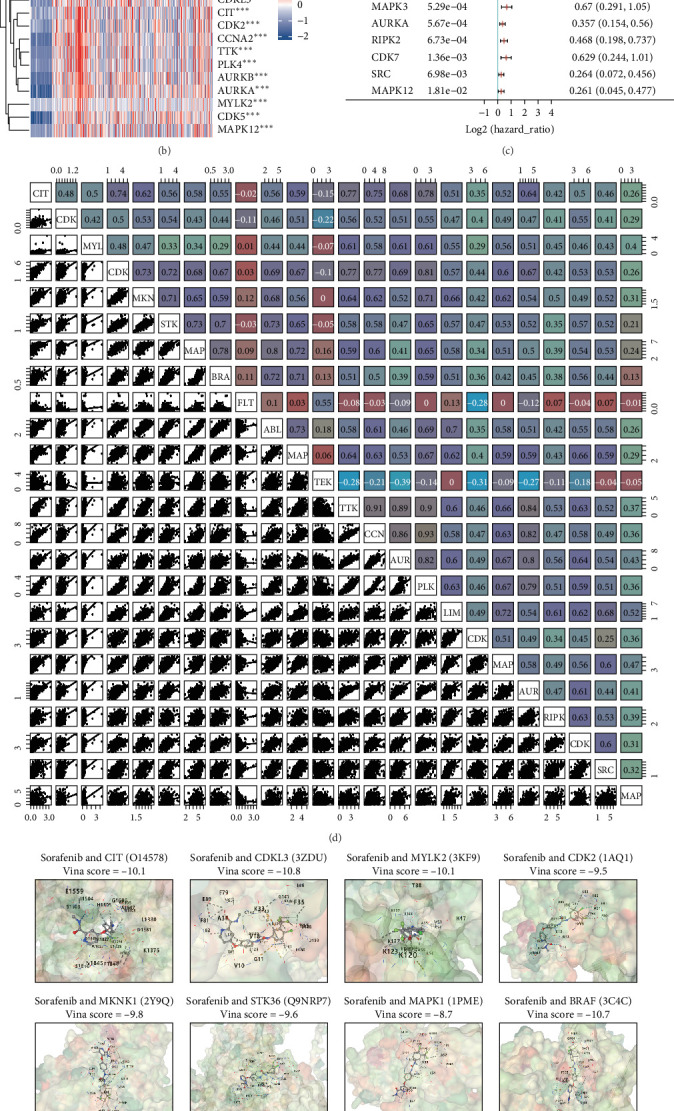
Identification of sorafenib targets within the TCGA-LIHC dataset. (A) Target prediction for sorafenib using the swisstargetprediction database. (B) Heatmap showing expression levels of sorafenib-related genes. (C) Forest plot depicting overall survival (OS) associated with these genes. (D) Correlation analysis among sorafenib-related genes. (E) Molecular docking results between sorafenib-related genes and sorafenib. *⁣*^*∗∗*^*p* < 0.01; *⁣*^*∗∗∗*^*p* < 0.001.

Sorafenib is a targeted therapeutic agent that markedly improves the prognosis of patients with advanced LIHC. To explore the mechanisms behind its anticancer activity, we employed the SwissTargetPrediction platform to identify potential sorafenib targets (Figure 1A). In total, 100 genes were predicted to be related to sorafenib; among these, 24 displayed differential expression in the TCGA-LIHC dataset when compared to adjacent non-cancerous tissues and were associated with overall survival (OS) outcomes (Figure 1B,C). Within this subset, two genes (FLT3 and TEK) acted as protective factors for OS, while the remaining 22 were classified as risk factors. Correlation analysis of these 24 genes in the TCGA cohort indicated that most exhibited positive relationships (Figure 1D). To further assess the binding precision of these targets, molecular docking was performed, confirming strong affinities between sorafenib and the candidate genes (Figure 1E).

### 3.2. Molecular Typing Based on Sorafenib-Related Genes

**Figure 2:**
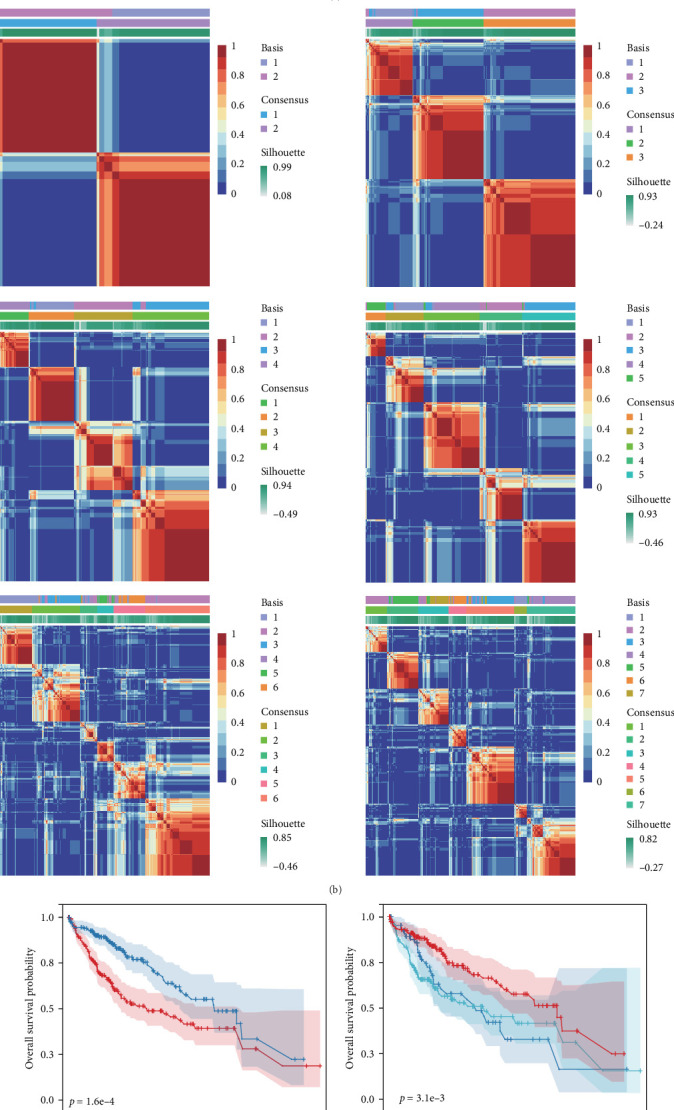
Clustering of sorafenib-associated genes. (A) Stability and performance assessment of clusters using multiple metrics. (B) Consensus matrix visualization from NMF analysis. (C,D) Survival outcome disparities among defined subgroups. (E,F) Differential expression patterns of sorafenib-response genes across clusters. *⁣*^*∗*^*p* < 0.05; *⁣*^*∗∗*^*p* < 0.01; *⁣*^*∗∗∗*^*p* < 0.001.

Leveraging expression data for genes associated with sorafenib response, samples within the TCGA-LIHC dataset were categorized using the non-NMF clustering method. To establish the optimal subgroup configuration for subsequent investigation, the consensus cumulative distribution function served as the principal selection metric. The point of maximum inflection (vertex) on these curves indicated the ideal number of clusters. Although the sharpest decline occurred at *k* = 7, generating seven subgroups was considered impractical for downstream analysis due to excessive fragmentation. Consequently, samples were stratified into two and three concurrent groupings for parallel evaluation (Figure 2A,B). Survival analysis demonstrated statistically significant disparities in OS across both dichotomous and tripartite classifications, with the most pronounced survival difference observed between the two-group division (Figure 2C,D). Expression profiles of the 24 sorafenib-related genes were further compared across the distinct groupings. Within the two-group stratification, every gene displayed statistically significant differential expression. Conversely, under the three-group classification, numerous genes failed to exhibit statistically significant expression variation between the subgroups (Figure 2E,F).

### 3.3. Analysis of the Correlation Between Sorafenib-Related Genes and Immune Infiltration

**Figure 3:**
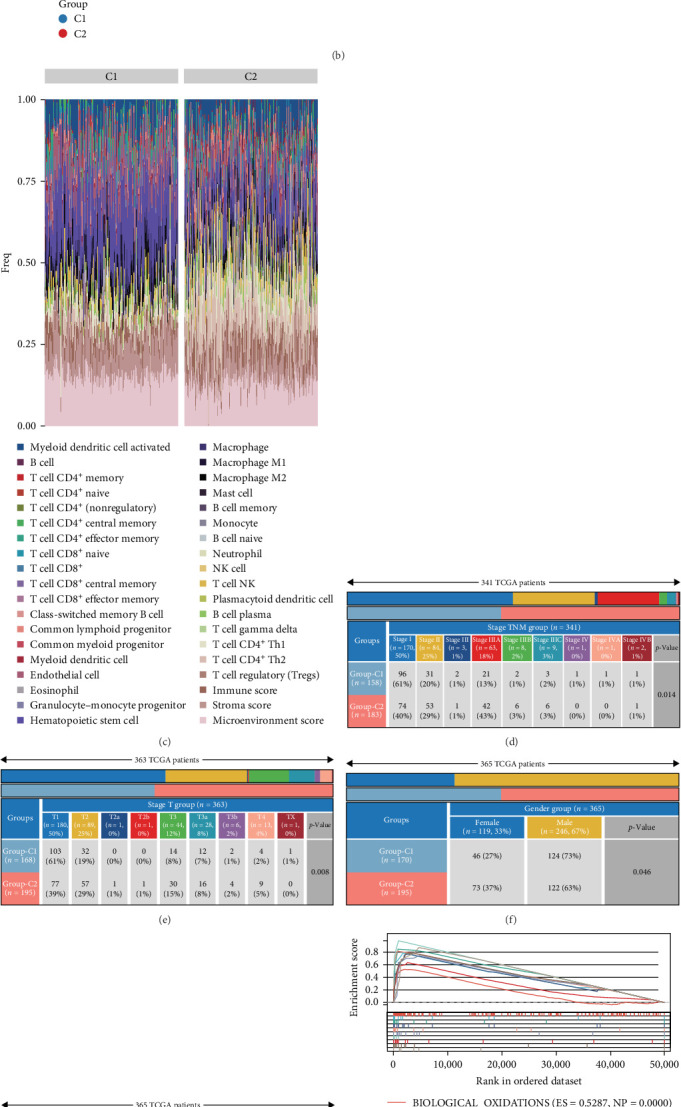
Association of sorafenib-associated genes with immune infiltration in LIHC. (A,B) XCELL computational framework analysis evaluating associations between sorafenib-responsive gene expression and immune infiltration levels. (C) Heatmap visualization demarcating immune infiltration landscapes across subgroups. (D–G) Demographic and clinicopathological distributions (T stage, TNM stage, gender, and survival status) within identified subgroups. (H,I) Functional enrichment characteristics distinguishing biological pathways between subgroups.

Immune cell infiltration within each TCGA-LIHC sample was quantified using signature genes corresponding to 35 distinct immune cell populations, identified via the XCELL computational method. Differential infiltration levels were observed for 19 immune cell types, indicating a potential association between sorafenib-responsive genes and the immune microenvironment in LIHC (Figure 3A,B). A heatmap depicted the contrasting immune landscapes between the two identified subgroups (Figure 3C). Furthermore, we analyzed the distribution of C1 and C2 subgroup patients across demographic and clinicopathological variables, including T stage, TNM stage, gender, and survival status, revealing significant patterns (Figure 3D–G). To explore potential mechanistic drivers, functional enrichment profiling was performed on the subgroups. This analysis revealed C1 was predominantly enriched in pathways involving biological oxidations, eicosanoid metabolism, and urea cycling, whereas C2 showed primary enrichment for processes related to cell cycle regulation, cellular senescence, and apoptosis (Figure 3H,I).

### 3.4. Constructing Prognostic Models

**Figure 4:**
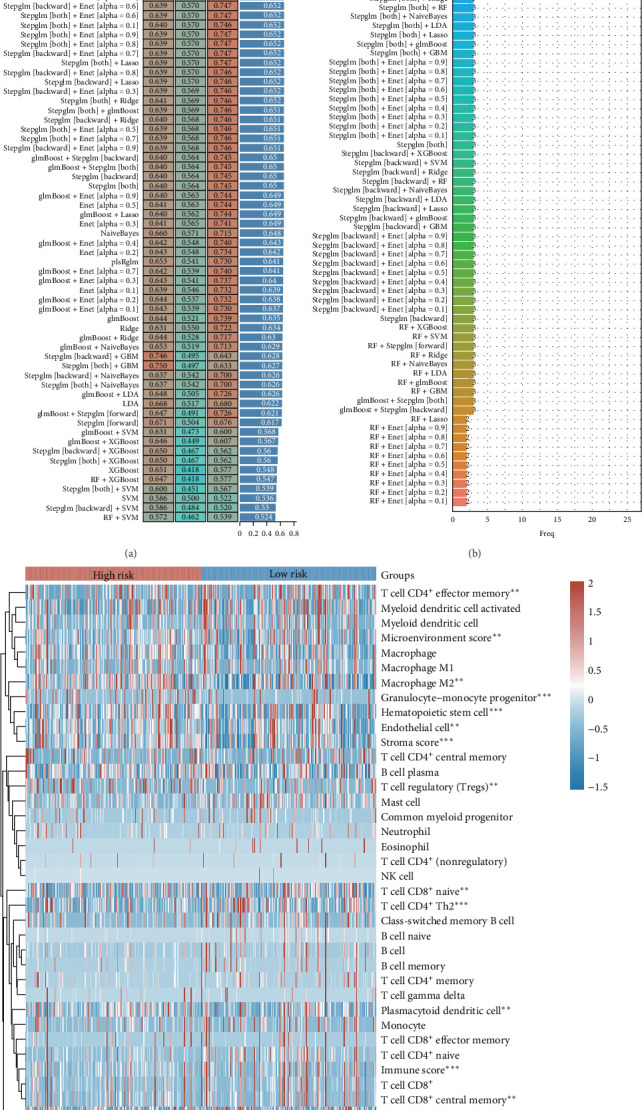
Build a prognostic model. (A) AUC values of prognostic models developed via combinations of different algorithms. (B) Number of genes incorporated into diagnostic models constructed with diverse algorithm combinations. (C) Variations in immune cell infiltration levels between high-risk and low-risk groups. (D,E) Distribution of patient numbers across stages in high-risk versus low-risk groups. *⁣*^*∗∗*^*p* < 0.01; *⁣*^*∗∗∗*^*p* < 0.001.

To evaluate the prognostic relevance of sorafenib-associated genes in LIHC, we integrated three independent cohorts: TCGA-LIHC, GSE76427, and ICGC. A multialgorithm framework was employed to develop a predictive model, revealing that the glmboost + RF method exhibited superior performance with a mean (AUC of 0.725 across all datasets (Figure 4A). This prognostic signature comprised eight pivotal genes: TTK, MYLK2, MAPK3, CCNA2, FLT3, MAPK12, CDK5, and RIPK2 (Figure 4B). Comparative analysis of immune infiltration in TCGA-LIHC revealed substantial disparities in 13 distinct immune cell populations between high- and low-risk subgroups (Figure 4C). Additionally, significant variations emerged in patient distribution across T stages and TNM stages when stratified by risk classification (Figure 4D,E). These results establish sorafenib-responsive genes as valuable prognostic biomarkers with significant implications for immune microenvironment regulation in LIHC.

### 3.5. MAPK12 as a Key Regulatory Gene for Sorafenib

**Figure 5:**
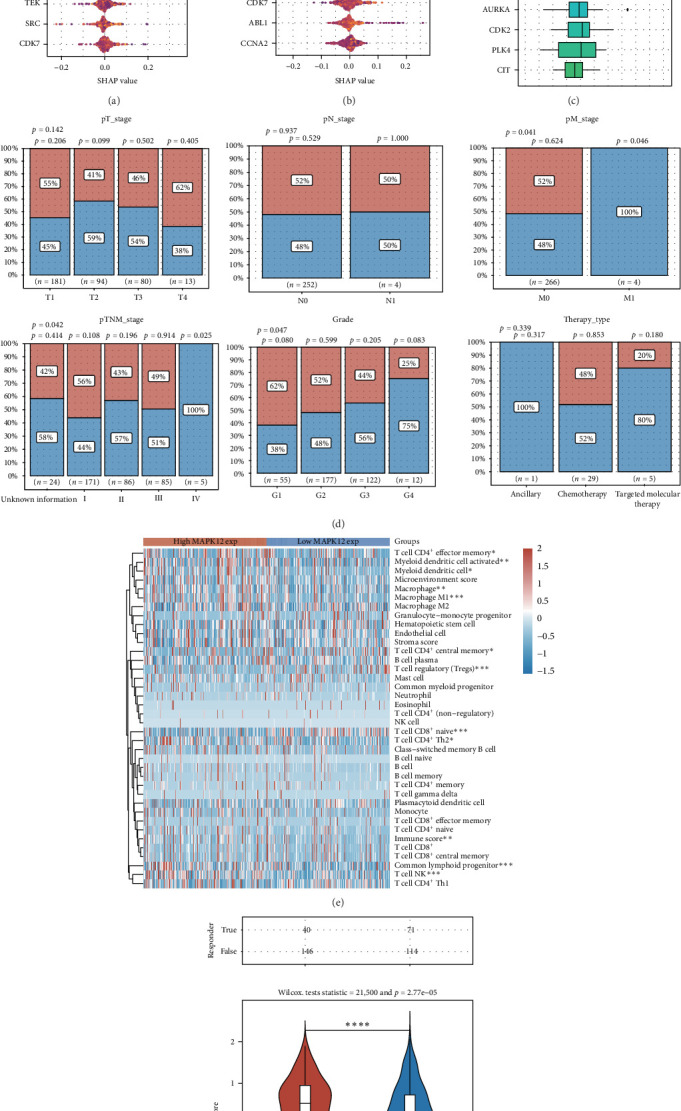
MAPK12 is the most significant gene among those related to sorafenib. (A,B) The analysis evaluates the prognostic relevance of sorafenib-related genes in patients with liver LIHC (LIHC) using the XgBoost algorithm. (C) The importance ranking of sorafenib-related genes is determined by the GOsemSim algorithm. (D) This panel displays the distribution of individuals with varying pathological parameters in the high and low expression groups of MAPK12. (E) A correlation analysis between MAPK12 and immune infiltration in LIHC is presented. (F) The relationship between MAPK12 and immune checkpoint therapy is analyzed using the TIDE algorithm. *⁣*^*∗*^*p* < 0.05; *⁣*^*∗∗*^*p* < 0.01; *⁣*^*∗∗∗*^*p* < 0.001; *⁣*^*∗∗∗∗*^*p* < 0.0001.

To identify key genes linked to sorafenib, we used the XGBoost algorithm to select the top 15 regulatory genes most closely associated with OS and progression-free survival (PFS) for patients in the TCGA-LIHC dataset. Additionally, we employed the GOsemSim tool to identify the top 15 core genes, with MAPK12 identified as the most prominent regulatory gene related to sorafenib (Figure 5A–C). Our analysis included multiple clinical variables—such as T stage, N stage, M stage, TNM stage, grade, and treatment regimens—to assess the distribution of patients with varying MAPK12 expression levels. Results showed a significant association between MAPK12 expression and M stage, TNM stage, and tumor grade (Figure 5D). TCGA-LIHC samples were stratified by MAPK12 expression (high vs. low), and differences in immune cell infiltration were evaluated—revealing significant variations across 11 immune cell types (Figure 5E). Moreover, patients with high MAPK12 expression demonstrated worse outcomes after immune checkpoint blockade (ICB) treatment (Figure 5F), implying that increased MAPK12 expression might correlate with reduced responsiveness to immunotherapy.

### 3.6. Functional Analysis of MAPK12 in LIHC

**Figure 6:**
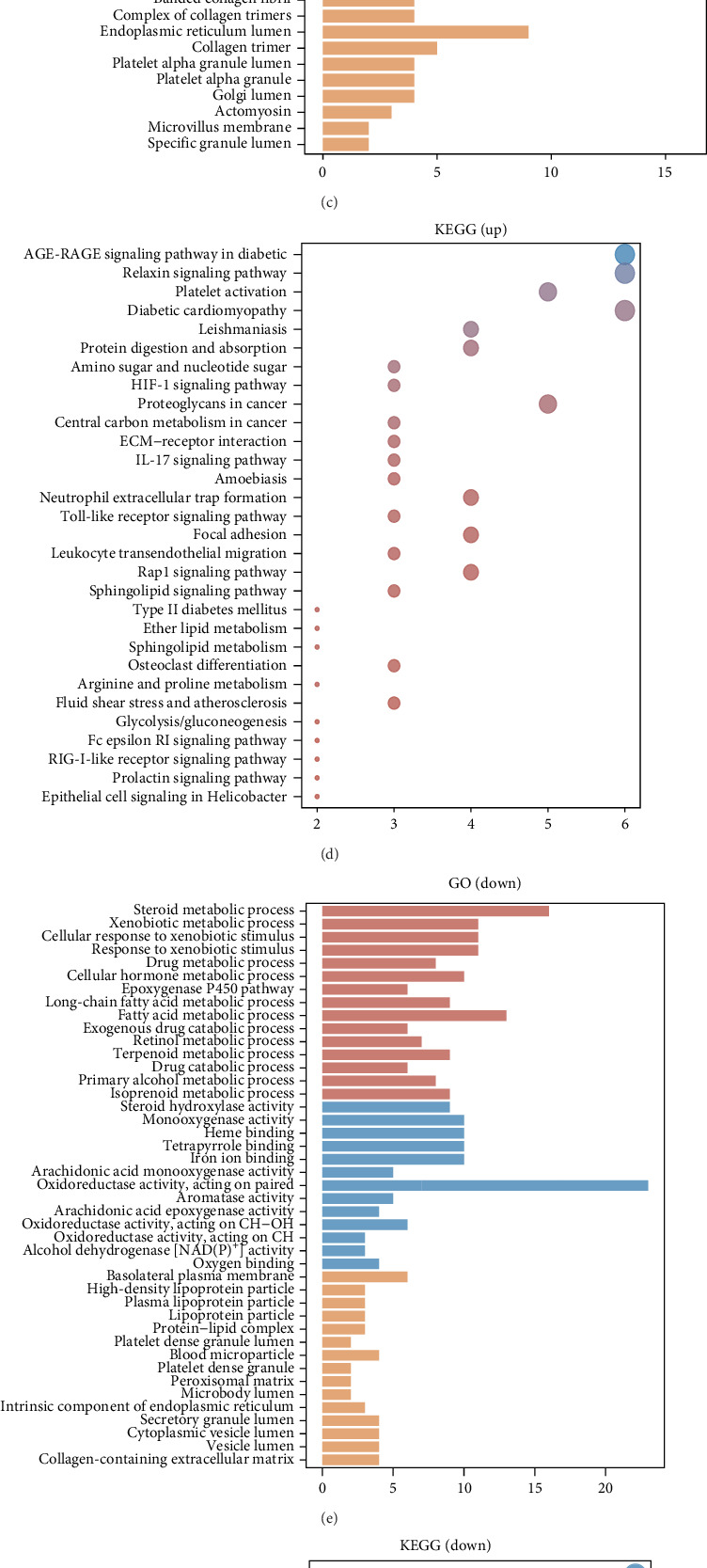
Functional analysis of MAPK12. (A,B) Difference analysis after grouping based on MAPK12 expression. (C–F) Analysis of MAPK12 functions by KEGG and GO methods. (G) Gene enrichment analysis of MAPK12.

In the TCGA-LIHC cohort, the median expression of MAPK12 was adopted as the classification threshold. Samples showing MAPK12 expression above this median were designated the high-expression group, whereas those with expression below the median comprised the low-expression group. Differential analysis employed stringent filters (*p* < 0.05; |Log2 fold change| > 2), with results visualized in expression heatmaps (Figure 6A,B). KEGG analysis indicated that genes upregulated in the high-MAPK12 group were enriched in pathways including HIF-1 signaling, diabetes-linked AGE-RAGE signaling, Relaxin signaling, diabetic cardiomyopathy, ECM-receptor interactions, and Toll-like receptor signaling. Conversely, downregulated genes correlated with metabolic processes like cytochrome P450-mediated xenobiotic/drug metabolism, retinol metabolism, steroid hormone biosynthesis, and bile secretion. GO analysis revealed upregulated gene involvement in extracellular matrix organization, structural components of the extracellular matrix, and collagen-rich matrices. Downregulated genes associated with steroid metabolism, oxidoreductase activity targeting paired donors, and basolateral plasma membrane localization (Figure 6). Given MAPK12′s strong HIF-1 pathway linkage, transcriptional regulation was explored, identifying significant HIF1A enrichment in the MAPK12 promoter region (Figure 6G).

### 3.7. MAPK12 Can Regulate the Stemness of LIHC Cells

**Figure 7:**
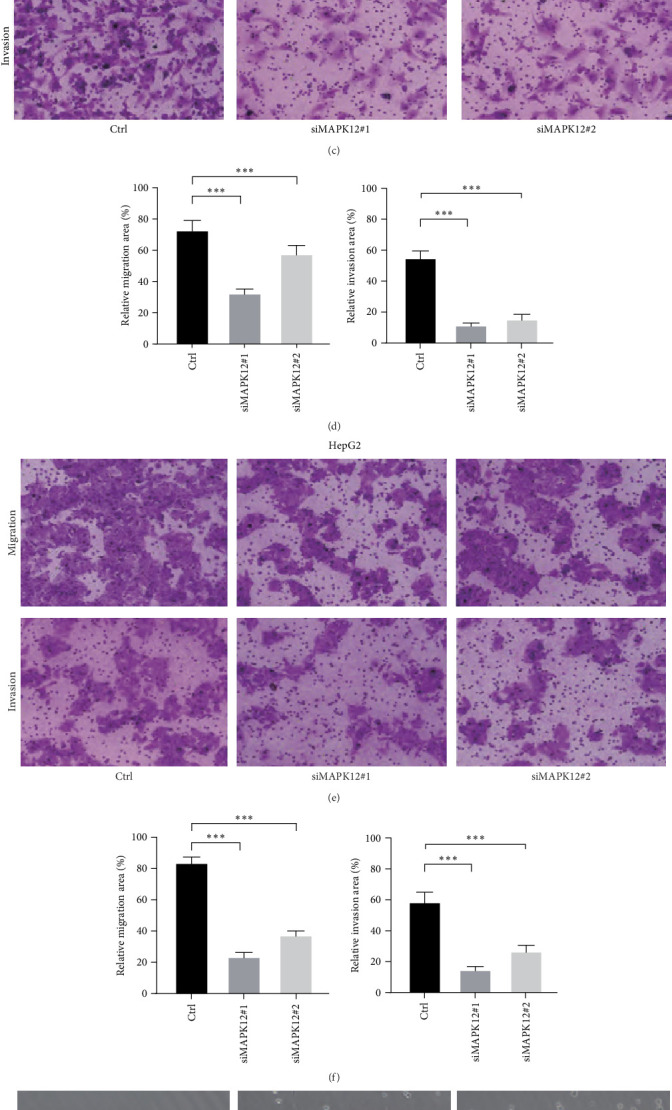
Knockdown of MAPK12 inhibits the stemness of liver cancer cells. (A) qRT-PCR was employed to assess the efficiency of MAPK12 siRNA knockdown. (B) A clone formation assay was conducted to evaluate the influence of MAPK12 knockdown on cell proliferation. (C–F) The effects of MAPK12 knockdown on cell migration were further analyzed using Transwell assays. (G,H) Knockdown of MAPK12 inhibits tumor sphere-forming ability. *⁣*^*∗∗∗*^*p* < 0.001.

An assessment was performed on the inhibitory effects of various target sites for MAPK12 siRNA by means of qRT-PCR techniques. The findings indicate that siMAPK12#1 and siMAPK12#2 exhibited the most pronounced inhibitory effects (Figure 7A). Consequently, these two target regions were selected for further cellular investigations. In the HCCLM3 and HepG2 cell lines, the silencing of MAPK12 resulted in a substantial decrease in cell proliferation (Figure 7B). Additionally, Transwell assays demonstrated that the knockdown of MAPK12 significantly reduced the migration and invasion capacities of both HCCLM3 and HepG2 cells (Figure 7). Considering that our gene enrichment analysis suggests an association between MAPK12 and the stem cell pathway in LIHC, we continued to investigate the effect of MAPK12 on tumor stemness through tumor sphere formation assays. Our results indicate that silencing MAPK12 has a substantial effect on tumor stemness (Figure 7G,H).

## 4. Discussion

LIHC patients typically confront a poor prognosis, chiefly attributed to the disease's substantial heterogeneity and intricate tumor microenvironment [28]. While existing biomarkers help assess prognosis and treatment responses, they often exhibit inadequate clinical sensitivity and specificity, limiting their value in guiding personalized immunotherapy. Consequently, discovering novel biomarkers is an urgent necessity. These markers would enable identification of high-risk individuals and yield critical insights into tumor immune landscapes, thereby, facilitating more precise therapeutic strategies. As tumor biology knowledge advances, researchers are anticipated to leverage multiomics data for uncovering LIHC-associated therapeutic targets, ultimately aiming to improve survival rates and quality of life.

Our study employed the SwissTargetPrediction database to pinpoint 100 potential sorafenib-binding genes, where RAF1 showed maximal binding affinity. This confirms the database's utility for drug target prediction. Established research indicates sorafenib functions as a multikinase inhibitor, primarily targeting Raf receptor tyrosine kinase pathways [29, 30]. Using TCGA-LIHC data, we discovered 24 differentially expressed genes with prognostic relevance in LIHC, including several validated disease regulators: Elevated CDK2 expression promotes LIHC proliferation [31], TTK inhibitors increase LIHC radiosensitivity via p21 signaling [32], and PLK4 overexpression correlates with adverse HCC prognosis [33]. To examine sorafenib-gene interactions, molecular docking was performed via CB-Dock2. Binding affinity was quantified by Vina scores (<−7 indicates strong binding). All 24 sorafenib-linked genes exhibited scores below −7, validating their selection [24]. Enrichment analysis revealed significant PD1/PDL1 checkpoint correlations and apoptotic pathway associations. ICI-based combination therapies have transformed LIHC treatment paradigms, substantially improving outcomes and initiating a new therapeutic era [34]. These regimens demonstrate superior efficacy versus sorafenib monotherapy for unresectable or advanced LIHC, particularly in initial treatment settings. Immune combinations achieve complete response rates exceeding sorafenib's by > 14-fold, suggesting potential cures in select advanced cases [35]. Our molecular insights may help clarify this clinical phenomenon.

Algorithms in machine learning are proficient at examining a wide array of biological datasets, such as genomic, transcriptomic, and clinical data. This capability enables researchers to derive fundamental insights from intricate datasets, thus, improving clinical relevance [36]. In this research, we utilized NMF cluster analysis—a particular technique in machine learning—to divide LIHC patients into two separate clusters [37]. The clinical traits of these clusters varied, with those in cluster 2 typically showing worse OS rates. Analysis of gene enrichment revealed that pathways related to cellular senescence and DNA damage repair were notably enriched in cluster 2, which could explain the reduced OS seen in these patients.

To facilitate early prognosis predictions in patients diagnosed with LIHC, we developed a prognostic model that employs various machine learning algorithms, which we subsequently validated across multiple datasets. Our results demonstrate that this model possesses substantial predictive accuracy. Moreover, through immunofluorescence analyses performed on 92 pairs of LIHC samples, we discovered a high expression level of MAPK12 in LIHC, indicating its possible utility as a predictive marker for diagnosing this condition. Furthermore, we evaluated the prognostic importance of MAPK12 in the same group of 100 patient samples, reinforcing its relevance in the prognosis of LIHC.

This research does have certain limitations, notably that our analytical outcomes largely rely on TCGA and associated databases, and there is a lack of an independent clinical cohort. Future studies should focus on incorporating a larger sample size or integrating an independent clinical cohort to corroborate our findings. Additionally, further investigations are required to confirm the role of MAPK12 in promoting stemness in LIHC cells.

## 5. Conclusion

This research represents the inaugural investigation into the impact of sorafenib-related targets on both the prognosis and immune infiltration in patients with LIHC. Notably, MAPK12 has been identified as the most crucial gene among these targets, indicating its promise as a new prognostic marker for individuals diagnosed with LIHC.

## Data Availability

The data generated or analyzed during the study are included in this article, and datasets are available from the corresponding author upon reasonable request.

## References

[1] Yousef E. H., El-Magd N. F. A., El Gayar A. M. (2023). Norcantharidin Potentiates Sorafenib Antitumor Activity in Hepatocellular Carcinoma Rat Model through Inhibiting IL-6/STAT3 Pathway. *Translational Research*.

[2] Wu B., Guo X., Wu Z., Chen L., Zhang S. (2025). COPB2 Promotes Hepatocellular Carcinoma Progression Through Regulation of YAP1 Nuclear Translocation. *Oncology Research*.

[3] Wei Q., Dai X., Wei J. (2024). Let-7i-5p Maintains the Stemness Via R-spondin2/Wnt Pathway in Hepatocellular Carcinoma. *Genes & Diseases*.

[4] Shao J., Zhao T., Liu J., Kang P. (2024). Targeting Liver Cancer Stem Cells: The Prognostic Significance of MRPL17 in Immunotherapy Response. *Frontiers in Immunology*.

[5] Xiao Z., Chen H., Xu N., Chen Y., Wang S., Xu X. (2024). MATR3 Promotes Liver Cancer Progression by Suppressing DHX58–Mediated Type I Interferon Response. *Cancer Letters*.

[6] Xi Q., Yang G., He X. (2024). M ^6^ A-Mediated Upregulation of lncRNA TUG1 in Liver Cancer Cells Regulates the Antitumor Response of CD8 ^+^ T Cells and Phagocytosis of Macrophages. *Advanced Science*.

[7] Poddar M. S., Chu Y.-D., Pendharkar G., Liu C.-H., Yeh C.-T. (2024). Exploring Cancer-Associated Fibroblast-Induced Resistance to Tyrosine Kinase Inhibitors in Hepatoma Cells Using a Liver-on-a-Chip Model. *Lab on a Chip*.

[8] Liu R., Liu Y., Zhang W. (2024). PCK1 Attenuates Tumor Stemness Via Activating the Hippo Signaling Pathway in Hepatocellular Carcinoma. *Genes & Diseases*.

[9] Hu D., Wang Y., Shen X. (2024). Genetic Landscape and Clinical Significance of Cuproptosis-Related Genes in Liver Hepatocellular Carcinoma. *Genes & Diseases*.

[10] Liang Y. (2024). Mechanisms of Sorafenib Resistance in Hepatocellular Carcinoma. *Clinics and Research in Hepatology and Gastroenterology*.

[11] Li X., Xu M., Shen J. (2022). Sorafenib Inhibits LPS-Induced Inflammation by Regulating Lyn-MAPK-NF-kB/AP-1 Pathway and TLR4 Expression. *Cell Death Discovery*.

[12] Guégan J.-P., Ezan F., Théret N., Langouët S., Baffet G. (2013). MAPK Signaling in Cisplatin-Induced Death: Predominant Role of ERK1 Over ERK2 in Human Hepatocellular Carcinoma Cells. *Carcinogenesis*.

[13] Wu R., Duan L., Cui F. (2015). S100A9 Promotes Human Hepatocellular Carcinoma Cell Growth and Invasion Through RAGE-Mediated ERK1/2 and p38 MAPK Pathways. *Experimental Cell Research*.

[14] He J., Wu F., Li J. (2024). Tumor Suppressor CLCA1 Inhibits Angiogenesis Via TGFB1/SMAD/VEGF Cascade and Sensitizes Hepatocellular Carcinoma Cells to Sorafenib. *Digestive and Liver Disease*.

[15] Eresen A., Zhang Z., Yu G. (2024). Sorafenib Plus Memory-Like Natural Killer Cell Immunochemotherapy Boosts Treatment Response in Liver Cancer. *BMC Cancer*.

[16] Yang L.-M., Wang H.-J., Li S.-L. (2024). Efficacy of Radiofrequency Ablation Combined With Sorafenib for Treating Liver Cancer Complicated With Portal Hypertension and Prognostic Factors. *World Journal of Gastroenterology*.

[17] Daina A., Michielin O., Zoete V. (2019). SwissTargetPrediction: Updated Data and New Features for Efficient Prediction of Protein Targets of Small Molecules. *Nucleic Acids Research*.

[18] Wang Z., Chen S., Li D. (2024). Integrative Analysis of Tumor Stemness and Immune Microenvironment Deciphers Novel Molecular Subtypes in Hepatocellular Carcinoma. *Genes & Diseases*.

[19] Yang Q., Gao L., Xu Y. (2024). Identification of TP53 Mutation-Associated Prognostic Genes and Investigation of the Immune Cell Infiltration in Patients With Hepatocellular Carcinoma. *Genes & Diseases*.

[20] Gaujoux R., Seoighe C. (2010). A Flexible R Package for Nonnegative Matrix Factorization. *BMC Bioinformatics*.

[21] Charoentong P., Finotello F., Angelova M. (2017). Pan-Cancer Immunogenomic Analyses Reveal Genotype-Immunophenotype Relationships and Predictors of Response to Checkpoint Blockade. *Cell Reports*.

[22] Liu L., Wang Y., Zhang Y. (2024). Gene Expression Profiles Contribute to Robustly Predicting Prognosis in Hepatocellular Carcinoma. *Genes & Diseases*.

[23] Feng S., Zhu L., Qin Y. (2024). Machine Learning Model Reveals the Role of Angiogenesis and EMT Genes in Glioma Patient Prognosis and Immunotherapy. *Biology Direct*.

[24] Liu Y., Yang X., Gan J., Chen S., Xiao Z. X., Cao Y. (2022). CB-Dock2: Improved Protein–Ligand Blind Docking by Integrating Cavity Detection, Docking and Homologous Template Fitting. *Nucleic Acids Research*.

[25] Chen H., Wu B., Guan K. (2025). Identification of Lipid Metabolism Related Immune Markers in Atherosclerosis Through Machine Learning and Experimental Analysis. *Frontiers in Immunology*.

[26] Yu G., Wang L.-G., Han Y., He Q.-Y. (2012). ClusterProfiler: An R Package for Comparing Biological Themes Among Gene Clusters. *OMICS: A Journal of Integrative Biology*.

[27] Dai F., Yuan Y., Hao J. (2024). PDCD2 as a Prognostic Biomarker in Glioma Correlates With Malignant Phenotype. *Genes & Diseases*.

[28] Gajos-Michniewicz A., Czyz M. (2024). WNT/*β*-Catenin Signaling in Hepatocellular Carcinoma: The Aberrant Activation, Pathogenic Roles, and Therapeutic Opportunities. *Genes & Diseases*.

[29] Wu Z.-C., Hui X.-G., Huo L. (2021). Antiproliferative Effects of Isoalantolactone in Human Liver Cancer Cells Are Mediated Through Caspase-Dependent Apoptosis, ROS Generation, Suppression of Cell Migration and Invasion and Targeting Ras/Raf/MEK Signalling Pathway. *Acta Biochimica Polonica*.

[30] Rudalska R., Harbig J., Snaebjornsson M. T. (2021). LXR*α* Activation and Raf Inhibition Trigger Lethal Lipotoxicity in Liver Cancer. *Nature Cancer*.

[31] Xin X., Lu Y., Xie S. (2020). MiR-155 Accelerates the Growth of Human Liver Cancer Cells by Activating CDK2 Via Targeting H3F3A. *Molecular Therapy - Oncolytics*.

[32] Zhang H., Yao W., Zhang M. (2021). TTK Inhibitor Promotes Radiosensitivity of Liver Cancer Cells Through p21. *Biochemical and Biophysical Research Communications*.

[33] Yeung S.-F., Zhou Y., Zou W., Chan W.-L., Ching Y. P. (2022). TEC Kinase Stabilizes PLK4 to Promote Liver Cancer Metastasis. *Cancer Letters*.

[34] Rizzo A., Ricci A. D., Gadaleta-Caldarola G., Brandi G. (2021). First-Line Immune Checkpoint Inhibitor-Based Combinations in Unresectable Hepatocellular Carcinoma: Current Management and Future Challenges. *Expert Review of Gastroenterology & Hepatology*.

[35] She M., Wu Y., Cheng M., Feng S., Li G., Rong H. (2024). Efficacy and Safety of PD-1/PD-L1 Inhibitor-Based Immune Combination Therapy Versus Sorafenib in the Treatment of Advanced Hepatocellular Carcinoma: A Meta-Analysis. *Frontiers in Medicine*.

[36] Li X., Yang Y., Xu S., Gui Y., Chen J., Xu J. (2024). Screening Biomarkers for Spinal Cord Injury Using Weighted Gene Co-Expression Network Analysis and Machine Learning. *Neural Regeneration Research*.

[37] Huang Z., Cai D., Sun Y. (2024). Towards More Accurate Microbial Source Tracking Via Non-Negative Matrix Factorization (NMF). *Bioinformatics*.

